# Utilization of preconception care and associated factors among pregnant women attending ANC in private MCH Hospitals in Addis Ababa, Ethiopia

**DOI:** 10.1186/s12884-023-05955-1

**Published:** 2023-09-08

**Authors:** Addisu Girma, Abera Bedada, Solomon Kumbi

**Affiliations:** Department of Obstetrics and Gynecology, Addis Ababa University, Addis Ababa, Ethiopia

**Keywords:** Preconception care, Utilization, Private MCH hospitals, Addis Ababa, Ethiopia

## Abstract

**Background:**

Despite its benefit in promoting maternal health and the health of her developing fetus, little is known about preconception care practice and its associated factors in Ethiopia. Moreover, preconception care utilization in private hospitals is not known. The purpose of this study, therefore, is to determine the utilization of preconception health care services and its associated factors among pregnant women following antenatal care in the private Maternal and Child Health hospitals in Addis Ababa.

**Methods:**

A Hospital based cross-sectional study was conducted from April 1 to April 30,2022 among 385 women attending ANC in private MCH hospitals. Bestegah and Hemen MCH hospitals were selected by convenience method. Data were collected by a pretested self-administered semi-structured questionnaire. To identify the factors associated with the utilization of preconception care, bivariable and multivariable logistic regression analysis were performed. Adjusted odds ratios with 95% confidence interval were estimated to assess the strength of associations, and statistical significance was declared at a p-value < 0.05.

**Results:**

The utilization of preconception care among the pregnant mothers according to our study was 40%. Professional/technical/managerial occupation (AOR = 4.3, 95%CI = 1.13, 16.33, P < 0.032), having good knowledge on preconception care (AOR = 3.5, 95%CI = 1.92, 6.53, P < 0.000), having unintended pregnancy (AOR = 0.1, 95%CI = 0.03, 0.42, P < 0.001), history of family planning use before conception (AOR = 3.9, 95%CI = 1.20, 12.60, P < 0.023), having pre-existing medical disease(s) (AOR = 8.4, 95%CI = 2.83, 24.74, P < 0.002), and having adverse pregnancy outcome(s) in previous pregnancies (AOR = 3.2, 95%CI = 1.55, 6.50, P < 0.000) were significantly associated with preconception care utilization.

**Conclusions:**

This study found out that the utilization of preconception care in the private MCH hospitals is still low i.e., only 40%. Occupation, level of knowledge, having unintended pregnancy, history of family planning use before conception, having adverse pregnancy outcome(s) in previous pregnancy and having pre-existing medical disease(s) were independently associated with preconception care utilization. Lack of awareness about the availability of the services and having an unintended pregnancy were the main reasons for not utilizing preconception care.

## Background

Preconception care(PCC) is defined as a set of interventions that aim to identify and modify biomedical, behavioral, and social risks to a woman’s health or pregnancy outcome through prevention and management [1]. Research has found that while interventions during pregnancy and childbirth are essential for improving outcomes, they are inadequate for realizing on going international improvements in perinatal health and achieving equitable outcomes for all people. The shift toward preconception health was put into motion over a decade ago to challenge this paradigm and focus attention, intervention, and support on the person before pregnancy [2].

Ethiopia has achieved remarkable success in reducing neonatal and maternal mortality in recent decades, but still has very high neonatal mortality rates (29 deaths per 1,000 live births) and maternal mortality ratios (412 deaths per 100,000 live births) [3]. One of the main reasons why maternal mortality is high in Sub-Saharan Africa is high rates of child marriage and unintended pregnancies apart from inadequate quality health care in time to address complications [4]. Ethiopia has one of the highest rate of teenage pregnancy 23.59% [5] and unintended pregnancy among reproductive age group 28% [6]. Therefore, there is little doubt that preconception care would make a significant improvement for our country in these regards [7].

Several studies conducted to look at the level of PCC across the world found the levels to be generally low. Utilization of preconception in China, Malaysia, and Sir Lanka is 40.0% [8], 44% [9], and 27.2% [10] respectively. Preconception care utilization in African countries like Nigeria and Ethiopia 10.3% [11], and 16.27% [12] respectively, which is much lower than the rest of the other developed countries. Moreover, preconception care utilization in private setup is generally not well studied. One study in Kenya found that preconception care utilization in private hospital was 35.% compared to 16.1% in rural public hospital [13].

Based on different articles findings, utilization of preconception care is influenced by age, gender, educational status, income, marital status, history of family planning use, health condition, history of ANC visit, parity, pregnancy intention, and gravidity [8–13].

In Ethiopia, several studies have been conducted to assess the utilization rate of preconception health care services among reproductive age women, pregnant and delivered women in community settings and public institutions, and the utilization rate was found to be low [12]. However, similar studies in private institutions are lacking. Hence, this study intends to determine the utilization of preconception health care services in private institutions.

## Methods

### Study setting and study period

A Hospital based study was conducted in two selected private MCH hospitals in Addis Ababa from April 1–30, 2022. As of 2018, the city has a total estimated population size of 7,823,600. Regarding health facilities and health services, there are 994 clinics, 99 health centers and 42 hospitals. Of the hospitals, 15 are registered public and 27 are registered private hospitals. Of the private hospitals, 8 are maternity specialty hospitals i.e., Maternal and Child Health hospitals.

### Study participants

**Source population** were all pregnant women who have been following ANC at Bestegah and Hemen MCH hospitals in Addis Ababa.

**Study population** were all sampled pregnant women who came for ANC at Betsegah and Hemen MCH hospitals in Addis Ababa during the data collection period.

### Exclusion criteria

Pregnant women who moved to Addis Ababa after conceiving were excluded from the study.

### Sample size determination

The sample size was calculated by using a single population proportion formula = Z2p(p-1)/d2 with assumptions of 35.1%, from Kenyan study [13] the population proportion of preconception care utilization in private institution, assuming 95% confidence interval, marginal error of 5% (0.05) and 10% non-response rate.

$$\begin{array}{l}n = \frac{{{Z^2}x(pq)}}{{{d^2}}}\\{\rm{p = 35}}{\rm{.1\% = 0}}{\rm{.351}}\,\,\,\,\,{\rm{q = 1 - p,}}\,{\rm{Z\alpha = 1}}{\rm{.96}}\,{\rm{and}}\,{\rm{d = 0}}{\rm{.05}}\\= \frac{{{{1.96}^2} \times 0.35(1 - 0.35)}}{{{{0.05}^2}}} = 350\end{array}$$

Adding 10% of no respondent participants; 350 + 0.1 × 350 = 385.

Secondly, factors associated with utilization of preconception care sample size was calculated using Open Epi Version 7 statistical software for two population proportions (Table 1).

**Table 1:** Second objective sample size calculation

Variables	Factors associated with utilization of PCC		CI	Power	OR	Sample size	References
	No	Yes					
Age > 30	41.9%	58.07%	95%	80	2.1	260	[14]
Educational status (Formal Education)	52.67%	48.33%	95%	80	5.5	73	[15]
Multiparity	47.60%	52.40%	95%	80	2.3	258	[14]
Good knowledge on PCC	49.10%	50.90%	95%	80	6.2	65	[16]

When reviewed many studies done in a different part of the world and in Ethiopia, most of them revealed that women’s education status, age of the women, multiparity and knowledge on PCC are the most determinant factors for utilization of preconception care.

After using the EPI-INFO version 7 to calculate the sample size using the above assumption with factors associated utilization of preconception care, maternal age was taken as it gives the maximum sample size i.e., 260 (by one-to-one ratio). Adding 10% non-response, the final sample size is 286.

By comparing the two sample sizes calculated using single proportion and double population formula, the larger sample size, which is 385 as the total sample for the study participants, was taken.

### Sampling technique and procedure

**Fig. 1:**
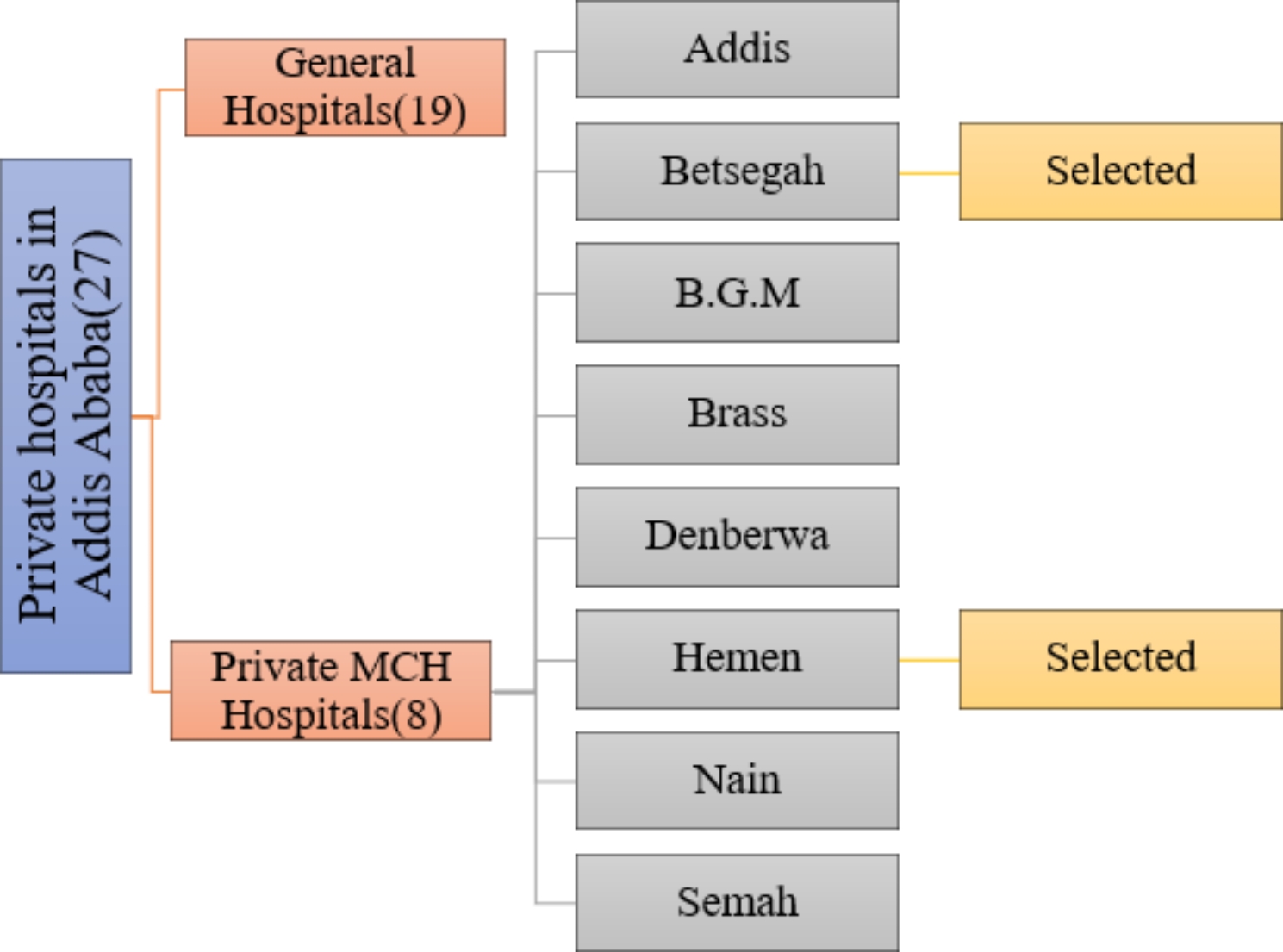
Flow diagram showing how the two private MCH hospitals were selected

Of the Private MCH hospitals, Betsegah and Hemen MCH hospitals were selected by convenience method (Fig. 1). In selected hospitals, all pregnant women attending antenatal clinic within the study period were recruited and participants were selected consecutively until the desired sample size was achieved to ensure that the entire population of antenatal attendees seen at each facilities who consented to participate were involved.

### Study variables

The dependent variable in this study was utilization of preconception care services among pregnant women following ANC. The independent variables were socio-demographic variables, previous adverse pregnancy outcome(s), preexisting medical disease(s), the knowledge level on preconception care, accessibility of health facilities, availability of preconception services, affordability of preconception care services and partner’s support.

### Operational definition and terms

#### Unintended pregnancy:

a pregnancy either mistimed or unwanted at a time of conception [6].

**Adverse Pregnancy outcome:** patient-reported history of one or more of the following outcomes in a previous pregnancy; preterm delivery, low birth weight, stillbirth, abortion or birth defect [17].

#### Cigarette smoking:

had a history of smoking or currently smoke regardless of amount [18].

#### Alcohol consumption:

intake of alcoholic drinks of any amount or type other than holidays and culturally special ceremony days [18].

#### Good knowledge:

those who have scored above or equal to 50% of the correct responses to preconception care knowledge questions [19].

#### Poor knowledge:

those who have scored less than 50% of the correct responses to preconception care knowledge questions [19].

#### Preconception care:

any interventions either advice or treatment, and lifestyle modification women received regarding components of preconception care before being pregnant [20].

#### Preconception care utilization:

if women received at least one type of intervention, either advice or treatment, and lifestyle modification care i.e., mentioned above at least once before being pregnant will be considered as mother utilized PCC.

#### Private MCH hospitals:

women’s and children’s specialty hospitals managed by individuals or groups, and which is not funded by the State, a public body or Non-governmental Organization.

### Data collection procedures and quality assurance

A data from pregnant woman following ANC were collected by a self-administered semi-structured questionnaire developed from previous published literature after modification to fit the research objective. The questionnaire was initially prepared in English and then translated into Amharic (local language) by different language experts of both languages and then to English to check its consistency. The questionnaire was used to elicit information regarding sociodemographic characteristics, knowledge about PCC, utilization of PCC prior to the current pregnancy and factors affecting the utilization of PCC. The questionnaires were administered by nurses. The questionnaire was pretested two weeks before the actual data collection with 5% of the sample size (20 pregnant women) at Zewditu Memorial Hospital in Addis Ababa, and the necessary amendments were done on the questionnaire per the pretest result. To minimize recall bias, the respondents were informed to provide information on events related to 3 months prior to the current pregnancy, and a calendar was provided to assist their recall. The overall activities of data collection were supervised and coordinated by the investigators. The collected data were checked for consistency, completeness, and relevance daily during the entire data collection by the principal investigator. After the data were collected from the respondents, it was translated back to English and analyzed using the statistical package SPSS version 26.

### Data processing and analysis

The collected data were entered to Statistical Package for Social Science (SPSS) version 26.0 for analysis. Descriptive statistics were done to describe the data. Binary logistic regression analysis was employed to examine the statistical association between utilization of preconception care and every single independent variable. Variables that showed statistical significance during bivariable analysis at (p-value < 0.25) were entered into multivariable logistic regression to identify statistically significant variables. Multicollinearity was tested by using the variance inflation factor and tolerance test. The Hosmer-Lemeshow test was used to check the model fitness for analysis, with a significance level of 0.310 indicating a good fit model. Adjusted odds ratios (AOR) with 95% CI were estimated to assess the strength of associations and statistical significance was declared at a p-value < 0.05. Tables, figures, and texts were used to present the results.

## Results

### Sociodemographic characteristics the study participants

Of the 385 participants studied, majority of the respondents (67.8%) were in the age group of 25–34 years, with the mean age and SD being 30.65 and ±4.87, respectively. Three-fourths of the participants completed tertiary level of education. More than 94.5% of them were married. The detailed sociodemographic characteristic are depicted in Table 2.

**Table 2:** The sociodemographic characteristics of study participants among pregnant women attending ANC in Betsegah and Hemen MCH hospitals in Addis Ababa, Ethiopia, 2022 (n-385)

Variables	Category	Frequency	Percent
Study Hospitals	Betsegah Maternity and Children Hospital	193	50.1
	Hemen Maternity and Children Hospital	192	49.9
Age	≤ 24	36	9.4
	25–34	261	67.8
	≥ 35	88	22.9
Educational status	Primary	26	6.8
	Secondary	72	18.7
	Tertiary	287	74.5
Religion	Orthodox	261	67.8
	Muslim	51	13.2
	Protestant	59	15.3
	Catholic	14	3.6
Marital Status	Single	12	3.1
	Married	364	94.5
	Divorced	9	2.3
Occupation	Professional/technical/managerial	152	39.5
	Sales and Services	197	51.2
	Skilled Manual	5	1.3
	Housewife	31	8.1
Monthly income	37.5-60USD^1^	13	3.4
	60-120USD	43	11.2
	120-390USD	234	60.8
	> 390USD	95	24.7

^1^ United States dollar

### Obstetric and medical characteristics of the study participants

Regarding obstetric characteristics, 56% of the participants were multiparous followed by nulliparous (40.5%). With regard to ANC booking, 93.5% of them booked at gestational age less than 16 weeks. The detailed obstetric and medical characteristics of study participants are depicted in (Table 3).

**Table 3:** Obstetric and medical characteristics of pregnant women attending ANC at Betsegah and Hemen MCH hospitals, Addis Ababa, Ethiopia, 2022 (n = 385)

Variables	Category	Frequency	Percent (%)
Pregnancy intention	Yes	229	59.5
	No	156	40.5
Parity	Nulliparous	156	40.5
	1–4	214	55.6
	Grandmultiparous (> 4)	15	3.9
GA^1^ at booking of current pregnancy	≤8 weeks	242	62.85
	12-16 weeks	118	30.65
	16 weeksand above	25	6.5%
Adverse pregnancy outcomes in the previous pregnancies	Yes	91	23.6
	No	294	76.4
Any adverse pregnancy outcomes in the previous pregnancies	Abortion	41	45.1
	Stillbirth	17	18.7
	Early neonatal death	5	5.5
	Congenital anomaly	9	9.9
	Preterm birth	11	12.1
	Others	8	8.8
Preexisting medical diseases	Yes	48	12.5
	No	337	87.5
Any preexisting medical diseases	Hypertension	22	45.8
	Diabetes	16	33.3
	Cardiac disease	5	10.4
	Others	5	10.4

^1^ Gestational Age

### Knowledge about preconception care

The findings of this study showed that, 58% (223) of the participants had good knowledge on preconception care. Concerning the specific knowledge, 91% had ever heard about preconception care. About 52.5% (202) of them mentioned screening for infectious disease like HIV, Syphilis, Hepatitis B virus as a components of preconception care. Furthermore, 76.4% (294) of the pregnant participants know that a woman should stop using alcohol and smoking cigarette before conception, and 68.1% (262) of them know that a woman should be on a healthy diet and use folic acid before conception (Table 4).

**Table 4:** Participants response for knowledge questions regarding preconception care among pregnant women of Betsegah and Hemen MCH hospitals, AA, Ethiopia, 2022 (n = 385)

Variables (Knowledge Questions)	Response	Frequency	Percent
Ever heard of preconception care	Yes	350	90.9
	No	35	9.1
Where did you hear about PCC	Health Facility	221	62.6
	In the community	45	12.7
	Mass media	80	22.7
	Internet	7	2
Family planning is a component of PCC	Yes	289	75.1
	No	96	24.9
Immunization is a component of PCC	Yes	210	54.5
	No	175	45.5
Screening for medical conditions; diabetes, hypertension, asthma, epilepsy	Yes	260	67.5
	No	125	32.5
Stopping use of environmental toxins; alcohol and cigarette smoking	Yes	220	57.1
	No	165	42.9
Lifestyle changes; healthy weight, healthy diet, and folic acid supplementation	Yes	214	55.6
	No	171	44.4
Screening for infectious disease; HIV, Syphilis, Hepatitis B virus	Yes	202	52.5
	No	183	47.5
A woman should be on family planning during preconception period	Yes	253	65.7
	No	132	34.3
A woman should be vaccinated before she conceives	Yes	137	35.6
	No	248	64.4
A woman should be screened for medical conditions like hypertension and diabetes	Yes	304	79.0
	No	81	21.0
A woman should stop using alcohol and smoking cigarette before conception	Yes	294	76.4
	No	91	23.6
A woman should a healthy weight, healthy diet and use folic acid before conception	Yes	262	68.1
	No	123	31.9
A woman should be screened for familial diseases	Yes	242	62.9
	No	143	37.1
A woman should be screened for infectious diseases like HIV^1^, HBV^2^, Syphilis and Gonorrhea	Yes	271	70.4
	No	114	29.6

^1^ Human Immunodeficiency Virus

^2^ Hepatitis B Virus

### Utilization of preconception care services

**Fig. 2:**
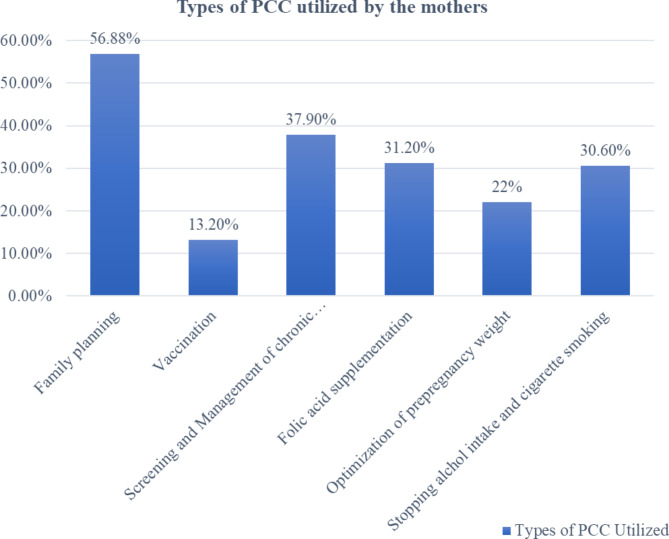
Types of preconception care services utilized by pregnant women of Betsegah and Hemen MCH hospitals, AA, Ethiopia, 2022 (n = 385)

In this study, 40% (154) of the study participants received preconception care before the current pregnancy. No significant differences were noted in the sociodemographic characteristics between the two MCH hospitals except for religion, the level of income, and previous adverse pregnancy outcome(s) (Table 5). The utilization of preconception care was almost similar between the hospitals i.e.,37.3% and 42.7% for Betsegah and Hemen MCH Hospital, respectively.The most frequently used types of preconception care service in this study was family planning 219 (56.88%) and the least utilized preconception service was vaccination 51(13.2%) (Fig. 2).

**Table 5:** Characteristics of study participants by the study hospitals, AA, Ethiopia, 2022 n = 385

Variables	Category	Study Hospitals		X^2^ test
		BMCH	HMCH	
Age	≤ 24	20	16	0.135
	25–34	137	124	
	≥ 35	36	52	
Education status	Primary	15	11	0.605
	Secondary	38	34	
	Tertiary	140	147	
Religion status	Orthodox	145	116	**0.009**
	Muslim	17	34	
	Protestant	23	36	
	Catholic	8	6	
Marital status	Single	5	6	0.552
	Married	181	183	
	Divorced	6	3	
	Widowed	1	0	
Occupation	Professional/Technical/Managerial	75	77	0.434
	Sales and Services	94	103	
	Skilled Manual	3	2	
	Housewife	21	10	
Monthly income	37.5-60USD (1955-3130ETB)	11	2	**0.000**
	60-120USD (3130- 6260ETB)	28	15	
	120-390USD (6260-20,335ETB)	131	103	
	> 390USD (> 20,335ETB)	23	72	
Parity	Nulliparous	71	85	0.327
	1–4	114	100	
	> 4	8	7	
Pregnancy intention	Yes	116	113	0.803
	No	77	79	
History of family planning use	Yes	111	108	0.802
	No	82	84	
Folic acid supplementation	Yes	52	68	0.073
	No	141	124	
GA at booking	Early Initiation(< 16weeks of gestation)	184	176	0.064
	Late Initiation (> 16weeks of gestation)	8	17	
Knowledge Level	Good	104	119	0.108
	Poor	89	73	
Previous adverse pregnancy outcomes	Yes	56	35	**0.013**
	No	137	157	
Preexisting medical diseases	Yes	26	22	0.550
	No	167	170	

Other findings in this study were 20.3% (78) of the pregnant were drinking alcohol before conception, of which forty-five of them still drinking alcohol until the end of the first trimester. Similarly, 1.8% [7] of the pregnant women were smoking cigarette before conception and five of them were still smoking cigarette until the end first trimester. On the contrary, only 30.6% (118) the pregnant women were advised on cessation of alcohol consumption and smoking cigarette during preconception period (Table 6).

**Table 6:** Utilization of preconception care services among pregnant women in Betsegah and Hemen MCH hospitals, AA, Ethiopia, 2022 (n = 385)

Variable	Response	Frequency	Percent
Family planning use before conception	Yes	219	56.9
	No	166	43.1
Vaccination before conception	Yes	51	13.2
	No	334	86.8
Screened for any medical conditions before conception	Yes	146	37.9
	No	239	62.1
Medical conditions screened for before conception	Hypertension	137	35.6
	Diabetes	124	32.2
	HIV	136	35.2
	Syphilis	94	24.4
	Anemia	112	29.1
Advised on the effects of alcohol and cigarette smoking on pregnancy	Yes	118	30.6
	No	267	69.4
Were you using alcohol before conception	Yes	78	20.3
	No	307	79.7
When did you stop using alcohol	2 months before conception	27	34.6
	2 months after conception	45	57.7
	Never	6	7.7
Were you using cigarette before conception	Yes	7	1.8
	No	378	98.2
When did you to stop using cigarette smoking	2 months before conception	2	28.6
	2 months after conception	5	71.4
When did you start using folic acid	Before conception	120	31.2
	After conception	100	26
	Never	165	42.9
Advised to have a healthy weight before conception	Yes	85	22.1
	No	300	77.9

### Partner support and heath facility

In this study 94% (362) of mothers make a decision about maternal health services with their partner. In addition, the cost of PCC services was considered fair by 75.3% (290) of the pregnant women. The most common reasons for not receiving PCC among the pregnant women who did not receive were lack of awareness about the availability of the services 64.8% (136) followed by because their pregnancy wasn’t expected 28.1% (65) and only 11.7% (27) did not acknowledge the importance of PCC (See Table 7).

**Table 7:** Health facility and partner support among pregnant women in Betsegah and Hemen MCH hospitals, AA, Ethiopia, 2022 (n = 385)

Variables	Category	Frequency	Percent
Decision maker regarding utilization of reproductive health services	Women	21	5.5
	Partner	2	0.5
	Joint	362	94
The type of support your partner offered during preconception	Accompanying to the health facility	269	69.9
	Financial support	234	60.8
	Psychological support	258	67
	Spiritual support	204	53
Were the following problems when you want to receive preconception care services	Long distance from health facility	99	25.7
	Availability of health care providers	58	15.1
	Perception of being low risk	57	14.8
	Transport money	38	9.9
	Religion	7	1.8
How affordable is the cost	Expensive	69	17.9
	Fair	290	75.3
	Cheap	26	6.7
If you did not receive any form of PCC services, what do you think is the reason	Not aware that the service is available	136	58.9
	The pregnancy was not expected	65	28.1
	It is not important before conception	27	11.7
	Others	3	1.3

### Factors associated with utilization of preconception care

The strength of association between independent variables and outcome variable (preconception care utilization) were measured using odds ratio and 95% confidence interval using binary logistics regression model. Accordingly, maternal age, educational status, marital status, occupation, monthly income, knowledge on preconception care, parity, use of family planning, unintended pregnancy, adverse pregnancy outcome(s) in previous pregnancy and preexisting medical condition had a p-value of ≤ 0.25 in the bivariable analysis and taken into the final model for multivariable analysis. In multivariable logistic regression analysis, occupation, knowledge on preconception care, use of family planning, unintended pregnancy, adverse pregnancy outcome(s) in previous pregnancy and preexisting medical condition(s) were significantly associated with utilization of PCC at p-value of ≤ 0.05 (Table 8).

**Table 8:** Bivariable and multivariable logistic regression analysis of factors affecting utilization of PCC among pregnant women having ANC in Betsegah and Hemen MCH hospitals, AA, Ethiopia, 2022 (n = 385)

Variable	PCC		P-value	COR (95%CI)	P-value	AOR (95%CI)
	Yes	No				
**Age**						
≤ 24	11	25	1		1	
25–34	100	161	0.369	1.4(0.67, 2.99)	0.690	0.8(0.26, 2.44)
≥ 35	43	45	0.065	2.2(0.95, 4.94)	0.656	0.7(0.23, 2.54)
**Education status**						
Primary	6	20	1		1	
Secondary	22	50	0.471	1.5(0.52, 4.15)	0.946	1.0(0.22, 4.99)
Tertiary	126	161	0.046	2.6(1.02, 6.69)	0.829	1.2(0.28, 4.91)
**Marital Status**						
Single	2	9	1		1	
Married	148	217	0.155	3.0(0.65,14.40)	0.115	1.9(0.25,14.05)
Divorced	4	5	0.214	3.6(0.48, 27.11)	0.176	1.5(0.07,31.41)
**Occupation**						
Professional/Technical/Managerial	77	75	0.003	4.3(1.66, 11.02)	**0.032**	**4.3(1.13, 16.33)**
Sales and Services	70	127	0.082	2.3(0.90, 5.86)	0.084	3.2(0.85, 12.03)
Skilled Manual	1	4	0.973	1.0(0.10, 11.01)	0.874	1.8(0.001,2592)
Housewives	6	25	1		**1**	
**Monthly Income**						
1955-3130ETB	3	10	0.111	0.33(0.08,1.29)	0.127	0.2(0.03, 1.54)
3130- 6260ETB	13	30	0.061	0.48(0.22,1.03)	0.692	1.2(0.40, 3.93)
6260-20,335ETB	93	141	0.205	0.73(0.45,1.18)	0.805	1.1(0.55, 2.17)
> 20,335ETB)	45	50	1			
**Level of knowledge**						
Good knowledge	124	99	0.000	5.5(3.42, 8.87)	**0.000**	**3.5(1.92, 6.53)**
Poor Knowledge	30	132	1		1	
**Parity**						
Nulliparous	68	87	1		1	
2–4	79	135	0.178	0.7(0.5, 1.14)	0.515	0.8(0.45, 1.50)
≥ 5	7	9	0.993	1.0(0.35, 2.8)	0.168	3.0(0.63,14.12)
**Pregnancy Intention**						
Yes	142	87	1	0.05(0.03, 0.10)	**1**	**0.1(0.03, 0.42)**
No	12	144	0.000		**0.001**	
**Family planning use before conception**						
Yes	140	79	0.000	19(10.4, 35.5)	**0.023**	**3.9(1.20,12.60)**
No	14	152	1		**1**	
**Adverse pregnancy outcome(s) in the previous pregnancies**						
Yes	60	31	0.000	4.1(2.50, 6.77)	**0.002**	**3.2(1.55, 6.50)**
No	94	200	1		1	
**Have any preexisting medical disease(s)**						
Yes	36	12	0.000	5.5(2.8, 11.10)	**0.000**	**8.4(2.83, 24.74)**
No	118	219	1		1	

Pregnant woman whose occupation is professional/technical/managerial were 4.3 times more likely to receive preconception care when compared to the housewives (AOR = 4.3, 95%CI = 1.13, 16.33, P < 0.032).

Those pregnant women who had good knowledge on PCC were 3.5 times more likely to utilize PCC than those having poor knowledge (AOR = 3.5, 95%CI = 1.92, 6.53, P < 0.000).

Pregnant mothers who had an unintended pregnancy were 90% less likely to utilize PCC compared to whose pregnancy were intended and those who were using family planning before conception were 3.9 times more likely to seek preconception care than mothers who weren’t using family planning before conception (AOR = 0.10, 95%CI = 0.03,0.42, P < 0.001).and (AOR = 3.9, 95%CI = 1.20, 12.60, P < 0.023).

Lastly, those having pre-existing medical disease(s) and adverse pregnancy outcome(s) in previous pregnancy were 8.4 and 3.2 times more likely to utilize PCC than those who had not (AOR = 8.4, 95%CI = 2.83, 24.74, P < 0.000) and (AOR = 3.2, 95%CI = 1.55, 6.50, P < 0.002).

## Discussion

Preconception care is an approach to optimize pregnancy outcomes which is crucial for many Sub-Saharan African countries, such as Ethiopia, where maternal and perinatal mortality remains alarmingly high.

This study found that the prevalence of preconception care utilization among the study participants was 40%. Our study findings showed higher utilization of preconception care compared to other community-based studies conducted in Ethiopia (Debre Birhan Town 13.4%, Mekelle City 18.2%, West Guji 22.3%) [15, 21, 22]. It also showed higher utilization of preconception care than studies done in Nigeria by Ekem et al. 10.3% and Adeyemo et al. 18.8% [11, 23], Siri Lanka by Patabendige M. et al. 27.2% [10]. The difference could be due to the different study setting and differences in the sociodemographic characteristics of the study participants. However; it was comparable with findings from China(40%) [8], Malaysia (44%) [9] and Kenya(35.1%) [13].

The most common types of preconception services utilized by the pregnant women was family planning 219 (56.88%) and the least utilized preconception service was vaccination 51(13.2%). This finding is different from a study done Mekelle city in which the most common utilized component of PCC was micronutrient supplementation (i.e., iron, folic acid) [22].

In addition, this study found that pregnant women whose occupation is professional/technical/managerial were 4.3 times more likely to receive preconception care when compared to housewives. This is consistent with the study done in France [24] and Nigeria [23]. This might be because of individuals working at professional/technical/managerial position are more likely to have better knowledge regarding preconception care and might have also better access to information than the housewives.

Awareness and knowledge were significantly associated with the utilization of preconception care in several studies [15, 22, 23, 25]. In this study, 90.9% (350) of the respondents had heard of preconception care. The main source of information was healthcare providers in 62.6% (221/), mass media in 22.7% (80), community in 12.7% (45) and internet 2% [7]. Similarly, 58% (223) of the participants had good knowledge on preconception care. This finding is different from studies done in Ethiopia [15, 18] and Nigeria [25] which showed lower awareness and knowledge about preconception care among pregnant women, which might be due the different study setting and different sociodemographic background of the study population.

In this study, pregnant women who had good knowledge about PCC were 3.5 times more likely to utilize PCC when compared to their counterparts who had poor knowledge about preconception care (AOR = 3.5, 95%CI = 1.92, 6.53, P < 0.000). This finding is consistent with two studies done in Ethiopia, Hosanna Town, which showed 82% reduced odds of utilizing preconception care among women, who had poor knowledge on preconception care than their counterparts [26] and West Guji which also showed 2.43 times odds of utilizing PCC among women having good knowledge compared to their counterparts [15]. Study in Malaysia [9],also showed positive association between good knowledge about preconception care and PCC utilization. This might be because good knowledge about preconception care helps them know about the importance of services. In addition to that, knowledge about PCC might improve attitudes towards PCC, which might in turn improve the utilization of preconception care.

Similarly, pregnant mothers who had an unintended pregnancy were 90% less likely to utilize PCC compared to whose pregnancy were intended and those using family planning before conception were 3.9 times more likely to seek preconception care than mothers who weren’t using family planning before conception. This finding is consistent with studies done in Hosanna Town [26] which showed that the odds of utilizing preconception care in women who had used family planning before current pregnancy were 2.45 times higher than those women who had not used family planning before current pregnancy.

Another study in Los Angeles [17] also showed among women with adverse pregnancy outcomes, having unintended pregnancy was associated with 70% lower odds of not utilizing preconception care. This is because those pregnant mothers who planned their pregnancy are more likely to visit a health facility to seek family planning services, hence, might have broader opportunities to receive preconception counselling services.

Furthermore, pregnant women having preexisting medical disease(s) and adverse pregnancy outcome(s)(s) in previous pregnancy were 8.4 and 3.2 times more likely to utilize PCC than those who had not. These findings are consistent with studies done in Malaysia [9],Los Angeles [17] and Mekelle City [22]. It can be assumed that these patients are more likely to have frequent health facility visits because of their medical conditions, hence, more likely to have adequate information regarding the effect of their medical diseases on pregnancy. In the same line, those pregnant mothers with previous adverse pregnancy outcome(s) are more likely to be concerned and might have heightened risk awareness compared to those who had not.

Moreover, about 75% of the study participants found no problem accessing health facilities. This is similar to studies done in Hosanna Town and West Guji where majority of women found no problem accessing health facilities [15, 26]. In addition, 75.3% of the pregnant women cited the cost of preconception care services as fair.

Lastly, among the pregnant mothers who did not receive PCC services, when asked their reasons for not receiving the care, two-thirds stated, they were not aware that the service was available, one-thirds stated their pregnancy was not expected and about 11.7% thought it was not important to them. This finding is similar to studies done in Los Angeles [17] which found that the common reason for not receiving PCC was not having a pregnancy intended. This shows that even though the awareness and knowledge is good and only few of them had poor attitude toward preconception care, only less than half utilized PCC services. Lack of awareness regarding the availability of the services and having unintended pregnancy could be the plausible explanation for the low utilization of preconception care in this study.

### Strength of the study

It is one of the very few studies conducted in Ethiopia in the area of PCC in private setting.

### Limitation of the study

Recall bias is a limitation of the study due to the nature of problem under the study requiring the potential ability of respondents to remember information retrospectively.

Selection bias is also a limitation as the study hospitals were chosen by the convenience sampling method.

The impact of husband’s educational attainment on PCC utilization was not included.

Certain components of preconception care, like optimization of psychological health, screening and management of intimate partner violence, and genetic counseling were not studied.

## Conclusions

This study found that the utilization of preconception care in the private MCH hospitals is still low i.e., only 40%. Occupation, level of knowledge, having unintended pregnancy, history of family planning use before conception, having adverse pregnancy outcome(s)(s) in previous pregnancy and having pre-existing medical condition(s) were independently associated with preconception care utilization.

Lack of awareness regarding the availability of PCC services and having unintended pregnancy were the obstacles for not receiving PCC.

### Recommendations

Health education regarding the importance and the availability of PCC services should be given to women of reproductive age in private MCH hospitals.

Interventions aimed at increasing the proportion of intended pregnancies will also be critical to promote the utilization of preconception care services in these hospitals.

Therefore, health education and providing information in the form of posters and displays about the components and importance of PCC in hospitals could potentially improve utilization of preconception care.

## Data Availability

The dataset generated during/or analyzed during the current study are not publicly available due to the papers written using this dataset have not been published but are available from corresponding authors on reasonable request.

## List of Abbreviations

AA: Addis Ababa
AKUH: Aga Khan University Hospital
ANC: Antenatal Care
AOR: Adjusted Odds Ratio
BMCH: Betsegah Maternity and Children Hospital
CI: Confidence Interval
HBV: Hepatitis B Virus
HIV: Human Immunodeficiency Virus
HMCH: Hemen Maternity and Children Hospital
GA: Gestational Age
IOM: Institute of Medicine
MCH: Maternal and Child Health
MLFH: Maragua Level Four Hospital
OR: Odds Ratio
PCC: Preconception Care
PI: Principal Investigator
SLE: Systemic Lupus Erythematosus
SPSS: Statistical Package for Health sciences
WHO: World Health Organization
